# Daphnoretin Induces Cell Cycle Arrest and Apoptosis in Human Osteosarcoma (HOS) Cells

**DOI:** 10.3390/molecules17010598

**Published:** 2012-01-09

**Authors:** Shoubin Gu, Jinhai He

**Affiliations:** Department of Orthopaedics, The Second Affiliated Hospital of Harbin Medical University, Harbin 150086, China

**Keywords:** daphnoretin, HOS, antiproliferative, cell cycle arrest, apoptosis

## Abstract

In this study antiproliferation, cell cycle arrest and apoptosis induced by daphnoretin in human osteosarcoma (HOS) cells were investigated. Antiproliferative activity was measured with the 3-(4,5-dimethylthiazol-2-yl)-2,5-diphenyltetrazolium bromide (MTT) assay. The IC_50_ value of daphnoretin was 3.89 μM after 72 h treatment. Induction of apoptosis was evidenced by apoptotic body appearance and Annexin V-FITC/PI apoptosis detection kit. Flow cytometric analysis indicated daphnoretin arrested the cell cycle in the G2/M phase. Western-blot assay showed that the G2/M phase arrest was accompanied by down-regulation of cdc2, cyclin A and cyclin B1. Moreover, daphnoretin inhibited Bcl-2 expression and induced Bax expression to desintegrate the outer mitochondrial membrane and causing cytochrome c release. Mitochondrial cytochrome c release was associated with the activation of caspase-9 and caspase-3 cascade. Our results demonstrated that daphnoretin caused death of HOS cells by blocking cells successively in G2/M phases and activating the caspase-3 pathway.

## 1. Introduction

Osteosarcoma (OS) is the most common, non-hematopoietic, primary malignant tumor of bone which occurs predominantly in adolescents and young adults [1]. It is a high grade neoplasm with rapid growth and early metastasis [2]. Even after the introduction of aggressive chemotherapy and wide excision of tumors, 30–50% of patients with initially localized disease subsequently experience recurrence, with subsequently poor clinical outcomes. Moreover, 20–30% of newly diagnosed cases present with metastatic disease [3,4]. Nowadays, chemotherapeutical drugs (methotrexate, *etc.*) are used in the treatment of osteosarcoma. However, it has been reported that osteosarcoma is only responsive to much higher doses of methotrexate [5,6]. This requirement for methotrexate in high doses for effectiveness may be explained by an intrinsic resistance of osteosarcoma to transport the drug across the cell membrane [7]. Thus, the discovery of new natural and synthetic products for osteosarcoma treatment is of great urgency.

Apoptosis is a form of programmed cell death which occurs through activation of cell-intrinsic suicide machinery [8]. It is a hallmark of anticancer drug-induced cell death [9,10,11]. Activation of the apoptotic cascade results from a complex interaction of molecular events [12]. It is characterized by condensation of cytoplasm and chromatin, DNA fragmentation, and cell fragmentation into apoptotic bodies, followed by removal and degradation of the dying cells by phagocytosis. Apoptosis is considered to be the major form of cell death in various physiological events [13,14].

Alterations in cell cycle protein/gene control have been identified in many malignant tumors, including osteosarcomas [15,16,17,18]. These proteins that regulate cell cycle progression at the critical checkpoint have been identified as important contributors to increased cell proliferation in certain human tumors [19,20,21,22,23]. Normally, progression through the cell cycle is governed by expression of proliferating cell nuclear antigen (PCNA), Ki-67, and by a family of cyclin-dependent kinases, which become activated by binding to cyclin proteins [16,18,23,24]. B-type cyclin-associated kinases regulate the G2/M transition [25]. Cyclin B1 appears in the cytoplasm of cells in the S-phase. It is transported to the nucleus at the G2/M transition and is broken down into anaphase by an ubiquitin-dependent pathway [26].

**Figure 1:**
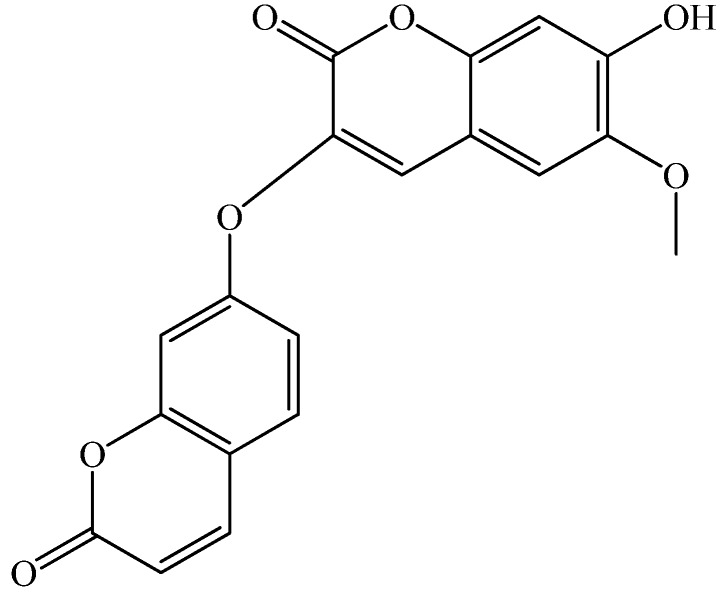
Chemical structure of daphnoretin.

*Stellera chamaejasme* L., belongs to the Thymealaeaceae, and is widely distributed in the northwest and southwest parts of China. The roots of *Stellera chamaejasme* L., can be used as a pesticide on bugs, flies and maggots, and can also control pests on crops, and pastures [27,28]. It has also been found that the methanol extract of the root of *Stellera chamaejasme* L. showed significant antitumor activities [29]. Chemical constituent investigations indicated *S. chamaejasme* L. is rich in dicoumarin and biflavonones which have been considered as being responsible for the beneficial effects of *S. chamaejasme* L. on human health [30,31]. Daphnoretin (Figure 1) is a natural dicoumarin constituent of *S. chamaejasme* L. with certain anti-HBV activity [32,33,34]. 

However, the anticancer activity of daphnoretin has not been elucidated yet. In the present study, we first attempted to evaluate antiproliferation activity of daphnoretin in human osteosarcoma (HOS) cells by MTT assay. The cell cycle arrest, apoptosis analysis were further studied by flow cytometry. The expression of cdc2, cyclin A and cyclin B1 was further evaluated by western blot. Morphological assessment of nuclear changes and measurement of mitochondrial membrane, Bcl-2, Bax, cytochrome c, caspase-3 and capspase-9 were used to assess apoptosis. 

## 2. Results and Discussion

### 2.1. Cytotoxicity Assays

The antiproliferative effect of daphnoretin was evaluated on three human osteosarcoma cell lines (HOS, U2-OS, MG-63) and normal human osteoblast cells using MTT assays. Taxol was used as positive control. The results were listed in Table 1. Daphnoretin exhibited stronger inhibition against HOS than U2-OS and MG-63. It has been suggested that both telomerase activity loss and sufficient telomere shortening are necessary to inhibit cell growth in telomerase positive osteosarcoma cells [35]. The result above prompt us that daphnoretin may inhibited telomerase activity in HOS (telomerase+) and MG-63 (telomerase+) than in U2-OS (telomerase-) cells which finally resulted in stronger cell growth inhibition. The further confirmation assay is urgently needed. Though the inhibition of taxol was stronger than that of daphnoretin against HOS, U2-OS and MG-63, its cytotoxicity was also much higher than that of daphnoretin. Thus, we can conclude that daphnoretin exhibits obvious antiproliferative effect on HOS.

**Table 1:** Inhibition concentrations 50% (IC_50_) values for daphnoretin towards HOS, U2-OS, MG-63 and normal human osteoblast cells determined by MTT assay. The symbol * indicates significant differences (*p* < 0.05) with respect to positive control (taxol). Results are represented means from three separate experiments.

Cell lines	IC_50_ (µM)	
	Daphnoretin	Taxol
HOS	3.89 *	3.01
U2-OS	8.72 *	5.35
MG-63	5.11 *	3.74
Normal human osteoblast cells	8.57 *	3.20

Many dicoumarins possess anti-tumor activity towards various human cancer cell lines, suggesting that they may be promising novel anticancer agent candidates [36,37]. Zhang *et al*. studied the anticancer activity of some novel dicoumarin derivatives (benzo[5,6]coumarin-3-carboxylic acid, triethylene-glycol dibenzo[5,6]coumarin-3-carboxylate, PEG (600) dibenzo[5,6]coumarin-3-carboxylate, 7-(*N,N'*-diethylamino)-coumarin-3-carboxylic acid, and triethylene-glycol di[7-(*N,N'*-diethylamino)]-coumarin-3-carboxylate) against SGC-7901 cells. All five of these dicoumarin derivatives showed certain anticancer activity, with IC_50_ values of 71.85 ± 1.26, 23.43 ± 2.27, 7.20 ± 7.13, 62.58 ± 1.29, and 11.45 ± 1.33 μg·mL^−1^, respectively [38]. Most of them showed low anticancer activity except benzo[5,6]coumarin-3-carboxylic acid. The above reports pointed out that the existence of methoxy and hydroxyl groups may contribute to the obvious anticancer activity of daphnoretin. The antitumor activity of dicoumarins or their structural derivatives need to be further evaluated in osteosarcoma cell lines.

### 2.2. Induction of Apoptosis by Daphnoretin

**Figure 2:**
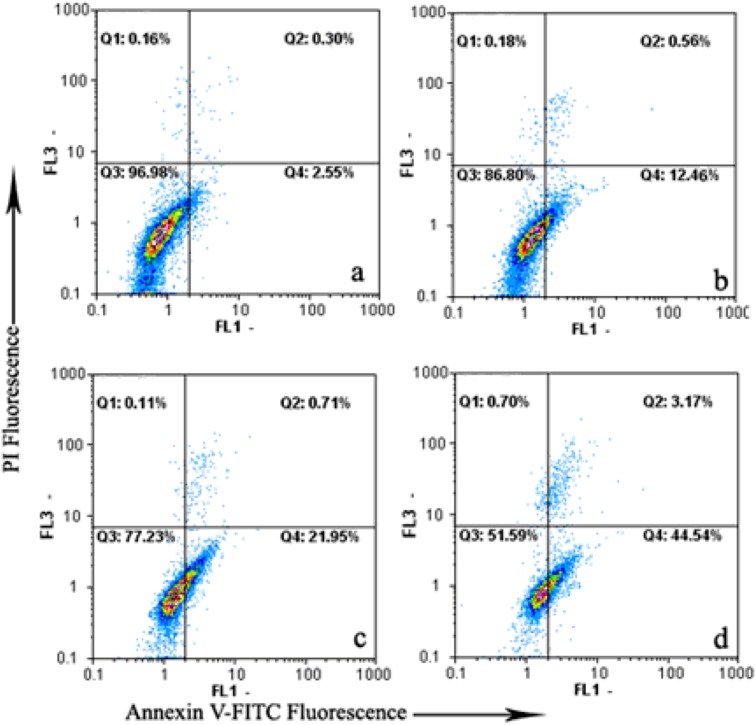
Daphnoretin-induced apoptosis in HOS using annexinV-FITC/PI. (a)-(d) Treatment with 0, 1, 2 and 4 μM daphnoretin for 48 h, respectively. The experiments were repeated three times and representative photographs are shown.

To determine whether daphnoretin-meditated inhibition of growth and proliferation was associated with apoptosis, the Annexin V-FITC/PI apoptosis detection kit was then used. As shown in Figure 2, only a small percentage of untreated HOS (2.85%) cells bound with annexinV-FITC. In contrast, the percentage of annexinV-FITC binding HOS cells significantly increased in a concentration-dependent manner after treatment with 1–4 μM daphnoretin (13.02% to 48.25%, *p* < 0.05). To summarize, data points were dispersed and shifted to the Q4 side in a dose-dependent manner when HOS cells were treated with daphnoretin, indicating that the cells moved to the early apoptotic stage. These experimental results demonstrate that daphnoretin induced apoptosis of HOS cells.

**Figure 3:**
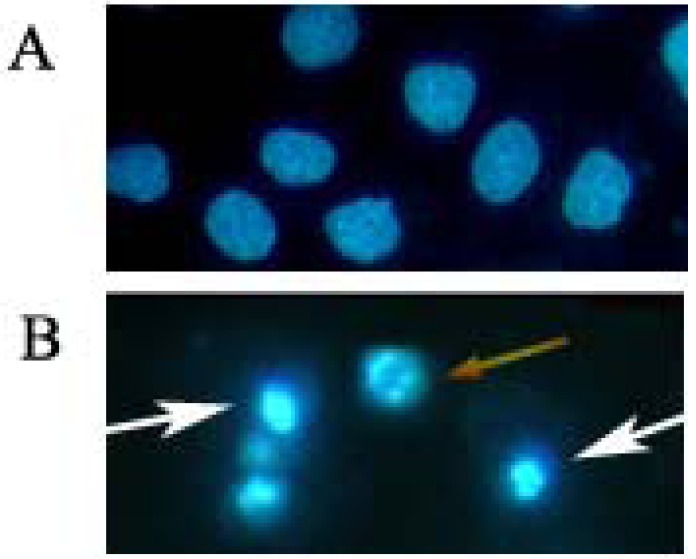
Morphological observation of HOS cells treated with 4 μM daphnoretin for 48 h by inverted fluorescence microscopy. Cells undergoing apoptosis and nuclear fragmentation are indicated by arrows. A, Untreated cells; B, daphnoretin-treated cells. The experiments were repeated three times and representative photographs are shown.

Hoechst 33258 staining was further used to investigate the affection of daphnoretin on nuclear morphology during cell apoptosis (Figure 3). The nuclei of untreated control HOS cells were stained in less bright blue and homogeneous color. By contrast, after treatment with 4 μM daphnoretin for 48 h, most cells exhibited very intense staining of condensed and fragmented chromatin. The white arrows pointed at the condensed chromatin. While the yellow arrow pointed at the fragmented chromatin which formed typical apoptotic bodies.

Apoptosis is a highly regulated death process by which cells undergo inducible non-necrotic cellular suicide. It plays an important role in anti-carcinogenesis [39]. Data obtained from Annexin V-FITC/PI assay and the appearance of apoptotic bodies showed that daphnoretin induced apoptosis in HOS cells.

### 2.3. Disruption of Δψ_m_ and Cytochrome c Release During Daphnoretin Induced Apoptosis

**Figure 4:**
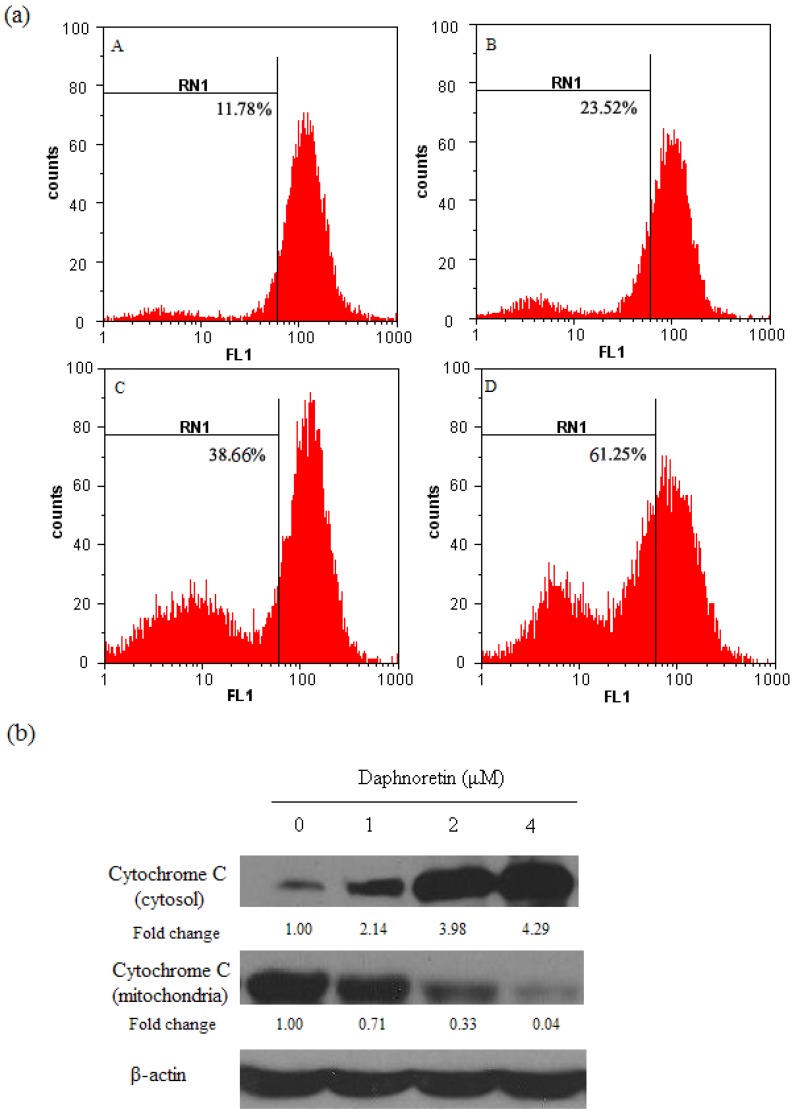
Disruption of Δψ_m_ and cytochrome c release during daphnoretin induced apoptosis. (**a**), Mitochondrial membrane potential of HOS cells after treatment with daphnoretin by using Rh123 staining. (A) Treatment with 0 µM daphnoretin; (B) treatment with 1 µM daphnoretin; (C) treatment with 2 µM daphnoretin; (D) treatment with 4 µM daphnoretin; The experiments were repeated three times and representative photographs are shown. (**b**), Immunoblotting for the expression of cytochrome c in response to treatment with daphnoretin (0, 1 µM, 2µM and 4µM) for 48 h. The blots were stripped and reprobed with anti-β-actin antibody to normalize the protein loading. Bands were quantitated by densitometric analysis. Fold change represents the protein level of the daphnoretin-treated cells relative to the control cells treated.

To assess the effects of daphnoretin on the mitochondrial apoptotic pathway, the Δψ_m_ in HOS cells treated with different concentrations of daphnoretin was measured using rhodamine 123 as fluorescent dye. As shown in Figure 4a, the number of cells with depolarized mitochondria increased with daphnoretin dose. The addition of daphnoretin at doses of 0–4 µM led to increasing percentages of Δψ_m_ disruption from 11.78% to 61.25% (*p* < 0.05).

During activation of mitochondrial-dependent apoptotic pathway, a number of signals can cause activation of the mitochondrial permeability transition and concomitant release of cytochrome c. Cytochrome c was greatly increased in the cytosol of cells treated with daphnoretin which indicating cytochrome c release from mitochondria to cytoplasm (Figure 4b). These results confirmed the involvement of mitochondria in daphnoretin-induced apoptosis.

### 2.4. Flow-Cytometric Analysis

**Figure 5:**
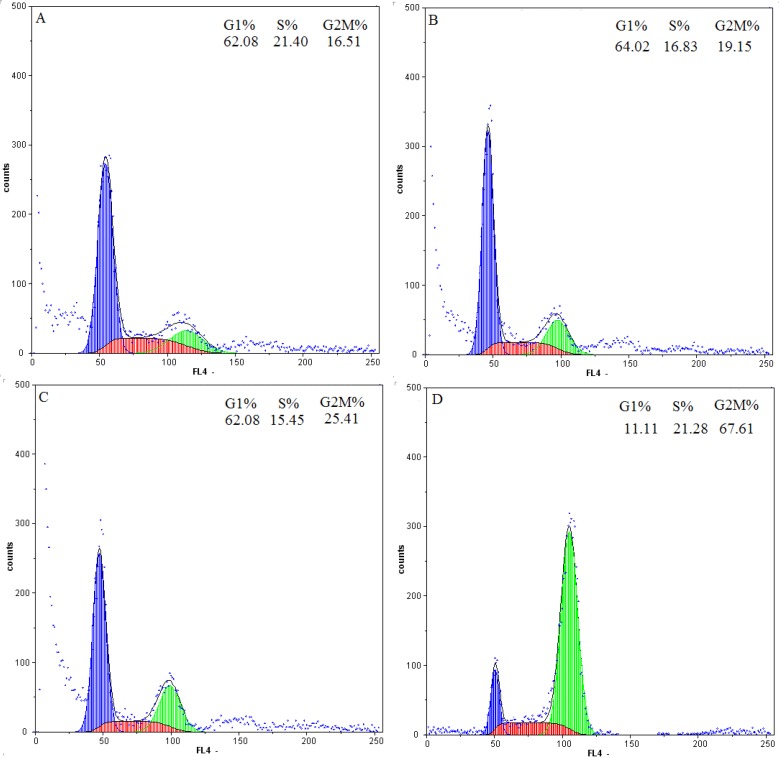
Flow cytometric analysis of daphnoretin-treated HOS cells for 48 h. A, HOS cells were treated with 0 μM daphnoretin. B, Cells were treated with 1 μM daphnoretin. C, Cells were treated with 2 μM daphnoretin. D, Cells were treated with 4 μM daphnoretin.

The DNA content was measured by flow cytometry to evaluate the cell cycle distribution of HOS cells with or without daphnoretin treatment. As shown in Figure 5, daphnoretin exerted growth-inhibitory effects via G2/M phase arrest in a concentration-dependent manner. Exposure to 1, 2, 4 µM daphnoretin could induce G2/M phase populati on increased from 19.15% to 67.61% (*p* < 0.05), compared to 16.51% of G2/M phase cells in untreated control samples. So we demonstrated that G2/M arrest followed by apoptosis is a mechanism of daphnoretin-induced HOS cells programmed death.

Cell cycle control is a major regulatory mechanism of cell growth [40]. Blockade of the cell cycle is considered as an effective strategy for the development of novel cancer therapies [41,42]. Cell cycle analysis of the treated culture revealed that daphnoretin induced a concentration-dependent G2/M phase cell cycle arrest with an accompaniment decrease in G1 and S phase. This confirmed that daphnoretin inhibited DNA synthesis and induced a block at the G2/M boundary. 

### 2.5. Effect of Daphnoretin on Cell Cycle-Related Proteins

**Figure 6:**
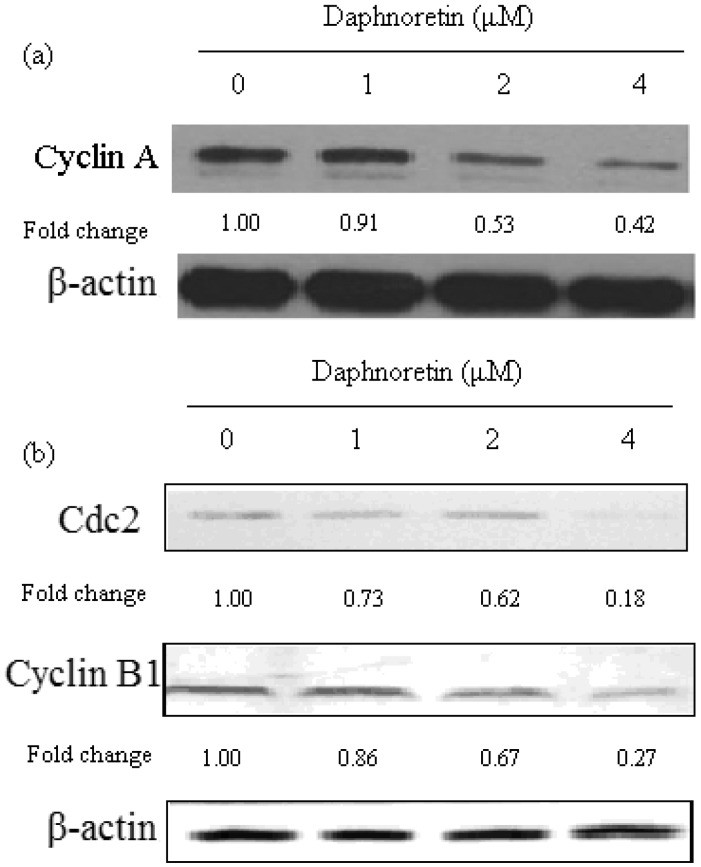
Immunoblotting for the expression of cell cycle-regulating proteins (a, cyclin A; b, cdc2 and cyclin B1) in response to treatment with daphnoretin (0, 1 µM, 2 µM and 4 µM) for 48 h. The blots were stripped and reprobed with anti-β-actin antibody to normalize protein loading. Fold change was calculated as described in Figure 4b.

Since cell cycle arrest was observed in daphnoretin-treated HOS cells by flow-cytometric analysis, it was of interest to test the effect of daphnoretin on cell cycle regulatory molecules (Figure 6). The level of cyclin A was significantly reduced in cells treated with daphnoretin in a dose-dependent manner. In addition, the level of cyclin B1as well as cdc2 also decreased after daphnoretin treatment in dose-dependent manner.

It is known that cell cycle dysregulation is a hallmark of anticancer drug-induced cell death. Regulation of proteins that mediate critical events of the cell cycle may be a useful antitumor target. Cyclin-dependent kinases (CDK) play an essential role in the regulation of cell cycle progression [43]. The levels of the CDK-activating proteins, cyclins, change during the cell cycle [44]. Among Cdks that regulate cell cycle progression, Cdk2 and Cdc2 kinases are primarily activated in association with cyclin A and cyclin B1 during the progression of the G2/M phase [45,46]. Thus, our data suggest that cell cycle arrest at the G2/M phase is mediated by reduction of cyclin A and cdc2/cyclin B complex formation, which is an essential step in regulating the cells passage into mitosis. It could be conclude the cell cycle arrest may partly explain apoptosis and anti-proliferative effects induced by daphnoretin.

### 2.6. Effect of Daphnoretin on Apoptosis-Related Proteins

**Figure 7:**
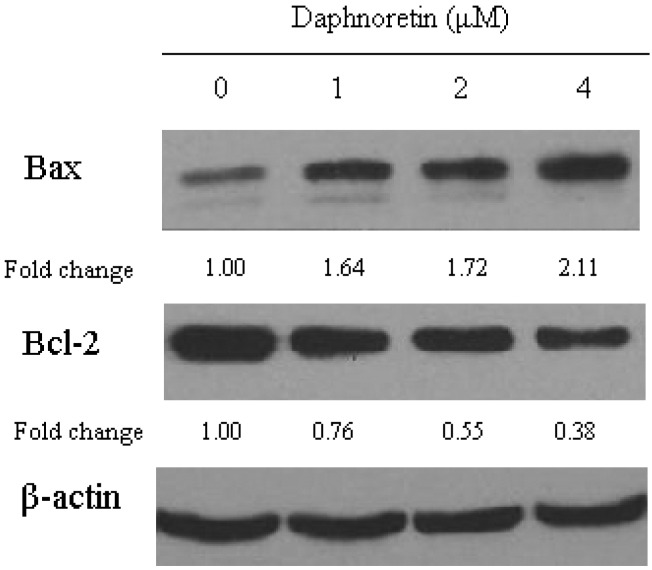
Immunoblotting for the expression of cell cycle-regulating proteins (Bax and Bcl-2) in response to treatment with daphnoretin (0, 1 µM, 2 µM and 4 µM) for 48 h.The blots were stripped and reprobed with anti-β-actin antibody to normalize protein loading. Fold change was calculated as described in Figure 4b.

**Figure 8:**
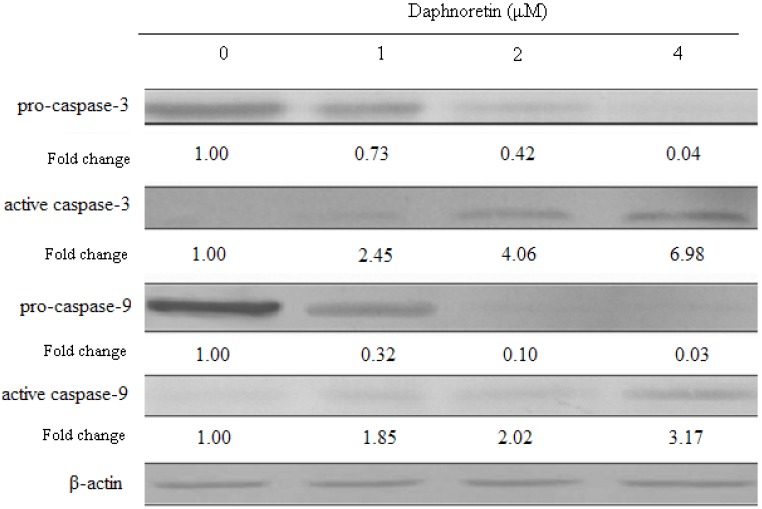
Changes in the expression of apoptosis-related proteins inresponse to treatment with daphnoretin for 48 h. The blots were stripped and reprobed with anti-β-actin antibody to normalize protein loading. Fold change was calculated as described in Figure 5b.

In order to investigate the mechanism by which daphnoretin induces apoptosis, the changes in the level of apoptosis-related proteins (Bax, Bcl-2, caspase-3 and caspase-9) were examined (Figure 7 and Figure 8). As shown in Figure 7, Western blot analysis revealed a significant increase in the expression of Bax in daphnoretin treated HOS cells, while there was a significant decrease in Bcl-2 expression, indicating that the Bax/Bcl-2 ratio increased significantly. A high Bax/Bcl-2 ratio was clearly correlated with increased apoptotic sensitivity to test reagents [47]. 

Pro-caspase-3 levels decreased upon treatment with daphnoretin, while the levels of active caspase-3 increased. Similarly, pro-caspase-9 levels decreased upon treatment with daphnoretin which induced increase active caspase-9.

It has been proven that cytochrome c and other pro-apoptotic factors are released from the mitochondria, allowing for formation of the apoptosome and subsequent cleavage and activation of caspase-9. Active caspase-9 then activates downstream caspases-3 and/or -7, which in turn cleave poly(ADP)-ribose polymerase (PARP) and other down-stream targets, resulting in apoptosis [48,49,50]. The activated caspase-3 and caspase-9 detected in the results further explained clearly the signaling pathway of daphnoretin-induced apoptosis in HOS cells.

The levels of cdc2, cyclin A and cyclin B1, decreased at daphnoretin treatment. Subsequent daphnoretin-induced apoptosis was observed. Moreover, daphnoretin inhibited Bcl-2 expression and induced Bax expression to desintegrate the outer mitochondrial membrane and causing cytochrome c release. Mitochondrial cytochrome c release was associated with the activation of caspase-9 and caspase-3 cascade. In conclusion, daphnoretin induced apoptotic cell death of human osteosarcoma (HOS) cells by blocking cells successively in G2/M phases and activating the caspase-3 and capspase-9 pathway.

## 3. Experimental

### 3.1. Materials

Daphnoretin and taxol were obtained from Sigma Chemical Co. (St. Louis, MO, USA) and was stored in glass vials with Teflon sealed caps at −20 ± 0.5 °C in the absence of light. A 10 mM stock solution of daphnoretin was prepared in dimethyl sulfoxide (DMSO) and stored at −80 °C. Deionized water was used in all experiments.

### 3.2. Growth of Cells

Human osteogenic sarcoma cell line (HOS, U2-OS, MG-63) and normal human osteoblast cells was cultured in Dulbecco’s modified Eagle’s media supplemented with 10% fetal bovine serum and 100 U/mL penicillin and 100 μg/mL streptomycin, HOS cell line was purchased from Harbin Medical University, China. The cells were kept at 37 °C in a humidified atmosphere containing 5% CO_2_.

### 3.3. Cytotoxicity Assays

Inhibition of cell proliferation by daphnoretin was measured by the MTT assay. Briefly, cells were plated in 96-well culture plates (1 × 10^5^ cells/well) separately. After 24 h incubation, cells were treated with daphnoretin (0, 0.5, 1, 2, 4, 8, 16 and 32 μM, eight wells per concentration) for 72 h, MTT solution (5 mg/mL) was then added to each well. After 4 h incubation, the formazan precipitate was dissolved in 100 μL dimethyl sulfoxide, and then the absorbance was measured in an ELISA reader (Thermo Molecular Devices Co., Union City, USA) at 570 nm. The cell viability ratio was calculated by the following formula: Inhibitory ratio (%) = (OD control − ODtreated) / ODcontrol × 100%. Cytotoxicity was expressed as the concentration of daphnoretin inhibiting cell growth by 50% (IC_50_ value).

### 3.4. Flow Cytometric Analysis of Apoptosis

The extent of apoptosis was measured through annexinV-FITC apoptosis detection kit (Beyotime Institute of Biotechnology, Shanghai, China) as described in the manufacturer’s instructions. After exposure to daphnoretin (0, 1, 2 and 4 μM) for 48 h, cells were collected, washed twice with PBS, gently resuspended in annexinV binding buffer and incubated with annexinV-FITC/PI in dark for 15 min and analyzed by flow cytometry using the FloMax software. The fraction of cell population in different quadrants was analyzed using quadrant statistics. The lower left quadrant contained intact cells; lower right quadrant apoptotic and in the upper right quadrant necrotic or post-apoptotic cells.

### 3.5. Morphological Observation of Nuclear Change

Morphological observation of nuclear change was assayed with Hoechst 33258. HOS cells (1 × 10^6^ cells/mL) were seeded in 6-well plates and treated with 4 μM daphnoretin for 48 h at 37 °C. Cells were collected, washed, fixed in 4% paraformaldehyde for 30 min and stained with 5 μg/mL Hoechst 33258 for 5 min at room temperature. The apoptotic cells were visualized using inverted fluorescence microscope (Nikon TE2000, Tokyo, Japan).

### 3.6. Cell Cycle Analysis

Cell cycle was assayed with CyStain [23]. Briefly, 1 × 10^6^ cells/well HOS cells were seeded in six-well plate and left for 24 h in incubator to resume exponential growth. Cells were exposed to daphnoretin (0, 1, 2 and 4 μM) and incubated for 48 h. Then, the cells were harvested and washed with PBS. After suspension in 800 μL PBS, 200 μL CyStain (Partec GmbH, Germany) was added to the mixture. The cell cycle distribution of 10,000 cells was recorded by flow cytometry (Partec), and the percentage of cells at G0/G1, S, and G2/M phases was analyzed with FloMax software. 

### 3.7. Changes of Mitochondrial Membrane Potential (Δψ_m_)

The uptake of the cationic fluorescent dye rhodamine 123 has been used for the evaluation of mitochondrial membrane potential [51]. HOS cells were seeded at 1 × 10^6^ cells/well into 6-well plates. After 24 h incubation, cells were treated with serial dilutions of daphnoretin (0, 1, 2 and 4 µM) for 48 h. Untreated controls and treated cells were harvested and washed twice with PBS. The cell pellets were then resuspended in 2 mL of fresh incubation medium containing 1.0 µM rhodamine 123 and incubated at 37 ◦C in a thermostatic bath for 30 min with gentle shaking and then analyzed by means of flow cytometry.

### 3.8. Western Blot Assay

HOS cells were treated with daphnoretin (0, 1, 2 and 4 µM) for 48 h, respectively. For isolation of total protein fractions, cells were collected, washed twice with ice-cold PBS, and lysed using cell lysis buffer [20 mM Tris pH 7.5, 150 mM NaCl, 1% Triton X-100, 2.5 mM sodium pyrophosphate, 1 mM EDTA, 1% Na_3_CO_4_, 0.5 μg/mL leupeptin, 1 mM phenylmethanesulfonyl fluoride (PMSF)]. The lysates were collected by scraping from the plates and then centrifuged at 10,000 rpm at 4 °C for 5 min. Protein from both the mitochondrial and cytosolic fractions was extracted as Soeda *et al.* [52]. Total protein samples (20 μg) were loaded on a 12% of SDS-polyacrylamide gel for electrophoresis, and transferred onto PVDF transfer membranes (Millipore, Billerica, MA, USA) at 0.8 mA/cm^2^ for 2 h. Membranes were blocked at room temperature for 2 h with blocking solution (1% BSA in PBS plus 0.05% Tween-20). Membranes were incubated overnight at 4 °C with primary antibodies (anti-caspase-3, anti-Bax, anti-Bcl2, anti- cytochrome c and β-actin was mouse monoclonal antibodies; anti-capspase-9, anti-cdc2 p34, anti-cyclin A and anti-cyclin B1 were rabbit monoclonal antibodies) at a 1:1,000 dilution (Santa Cruz Biotechnology, Santa Cruz, CA, USA) in blocking solution. After thrice washings in TBST for each 5 min, membranes were incubated for 1 h at room temperature with an alkaline phosphatase peroxidase-conjugated anti-mouse secondary antibody at a dilution of 1:500 in blocking solution. Detection was performed by the BCIP/NBT Alkaline Phosphatase Color Development Kit (Beyotime Institute of Biotechnology) according to the manufacturer's instructions. Bands were recorded by a digital camera (Canon, EOS 350D, Tokyo, Japan).

### 3.9. Statistical Analysis

The Student’s t-test was used to compare the difference between two different groups. A value of *p* < 0.05 was considered to be statistically significant.

## 4. Conclusions

In summary, the present study showed that daphnoretin inhibited the growth of HOS cells and the reduction in cell viability resulted from cell cycle arrest at G2/M phase, accompanied by the levels of cdc2, cyclin A and cyclin B1 decreased after daphnoretin treatment. Moreover, daphnoretin-induced HOS cells apoptosis by mitochondria-dependent pathway, involving inhibition of Bcl-2 expression and induction of Bax expression to disintegrate the outer mitochondrial membrane and to cause cytochrome c release. Further downstream of the apoptosis cascade, daphnoretin activated caspase-9 and caspase-3. All these evidences provide a rationale to explore daphnoretin as a preventive and perhaps as a chemotherapeutic agent in the management of osteosarcoma.References 
